# A Time Series Study of *Lophelia pertusa* and Reef Megafauna Responses to Drill Cuttings Exposure on the Norwegian Margin

**DOI:** 10.1371/journal.pone.0134076

**Published:** 2015-07-28

**Authors:** Autun Purser

**Affiliations:** Oceanlab, Jacobs University, Bremen, Germany; University of Kansas, UNITED STATES

## Abstract

As hotspots of local biodiversity in the deep sea, preservation of cold-water coral reef communities is of great importance. In European waters the most extensive reefs are found at depths of 300 – 500 m on the continental margin. In Norwegian waters many of these reefs are located in areas of interest for oil and gas exploration and production. In this study drilling was carried out in the Morvin drill field in proximity to a number of small *Lophelia pertusa* coral reefs (closest reefs 100 m upstream and 350 m downstream of point of waste drill material release). In a novel monitoring study, ROV video surveys of 9 reefs were conducted prior, during, immediately after and >1 year after drilling operations. Behavior of coral polyps inhabiting reefs exposed to differing concentrations of drill cuttings and drilling fluids (waste drilling material) were compared. Levels of expected exposure to these waste materials were determined for each reef by modelling drill cutting transport following release, using accurate in-situ hydrodynamic data collected during the drilling period and drill cutting discharge data as parameters of a dispersal model. The presence / absence of associate reef species (*Acesta excavata*, *Paragorgia arborea* and *Primnoa resedaeformis*) were also determined from each survey video. There were no significant differences in *Lophelia pertusa* polyp behavior in corals modelled to have been exposed to pulses of >25 ppm drill cutting material and those modelled to be exposed to negligible concentrations of material. From the video data collected, there were no observed degradations of reef structure over time, nor reductions of associate fauna abundance, regardless of modelled exposure concentration at any of the surveyed reefs. This study focused exclusively on adult fauna, and did not assess the potential hazard posed by waste drilling material to coral or other larvae. Video data was collected by various ROV’s, using different camera and lighting setups throughout the survey campaign, making comparison of observations prior, during and post drilling problematic. A standardization of video monitoring in future monitoring campaigns is recommended.

## Introduction

Cold-Water Coral (CWC) reefs are found throughout the oceans of the world [1,2,3]. As at tropical coral reefs, scleractinian cold-water coral fauna construct complex three dimensional structures over generations by secreting calcium carbonate skeletons as they grow [4,5]. In contrast with many tropical reef scleractinian species, algal zooxantellate symbiotic partners are not associated with CWC species, and therefore distribution may extend to depths below the photic zone. Lack of a symbiotic partner, whilst allowing for greater depth distribution, limits reef establishment to areas where sufficient food is delivered to the seafloor for growth, either in pulses of fresh surface primary production or high volumes of refractory material transported in the bottom ocean layers [6,7,8,9,10]. Further environmental factors, such as temperature range, flow velocity and substrate type also influence distribution [reviewed in 2].

Over the last two decades published research has described CWC reefs as hotspots of high local biodiversity [11,12,13]. Although few species found at CWC reefs are endemic, species richness and ecosystem biomass are higher within and in the vicinity of reefs than in comparably sized areas of the surrounding seafloor [12,14,15].

Commonly found at depths associated with the continental shelf edge, (300–500m depth), CWC reefs have historically been subjected to mechanical damage from fishing [16, 17, 18]. Progressively, such direct impacts are being legislated against throughout European seas [19, 20, 21, 16], although the consequences of increased particulate exposure following resuspension of adjacent seabed sediments by bottom trawling are as yet uncertain [16, 22]. There is also concern over the potential hazards threatening CWC ecosystems resulting from the activities of the offshore oil and gas industry [22]. During the last 30 years this industry has progressively increased its operational depth range, and there is interest in extracting oil and gas from strata underlying seafloor supporting CWC reefs. There have been industry funded attempts to gauge exactly how much impact drilling operations may have on benthic fauna [23, 24, 25, 26, 27, 28, 29], and CWC reefs in particular [30, 31, 22, 32, 33]. The direct spatial footprint of drilling operations is small, with the drill shaft and drill well impacting on only a few square meters of seabed and the drill rig anchors impacting on areas of a few hundred square meters [29]. The main environmental concerns posed by the drilling process are those relating to the release of waste material to the ocean during drilling operations. Such releases may cause changes in seafloor surface composition for considerable distances from point of release [34], potentially also smothering benthic fauna or interfering with the feeding ability of filter feeders [35, 36, 37, 31, 29, 33].

It is within Norwegian waters that the most expansive European CWC reef structures are found today [38]. Complexes such as the Røst and Sula Reefs are many kilometers in length, running parallel with the edge of the continental margin on large ridge crests associated with the Storegga landslides, with reef development commencing ~8,000 yrs B.P. [39]. Elsewhere on the Norwegian shelf reefs are commonly smaller in size, consisting of arrays of small ridge mounds of <150 m diameter, such as the Traena Reef [13, 22]. In Norwegian waters a precautionary principle is required for drilling operations by legislating bodies (Activities regulations §§ 52-54(http://www.ptil.no/activities/category399.html#_Toc345662833)) [40].

As a filter feeding sessile species, *Lophelia pertusa*, the key habitat forming scleractinian coral on the Norwegian margin (citations in [41]) cannot actively avoid exposure to settling particulates or those in suspension. Exposure to high concentrations of particles has been shown to be detrimental to warm water coral species, as a consequence of light depravation for algal symbiotic species as well as from increased surface anoxia and subsequent bacterial attack following burial smothering [42, 43, 44]. The lack of an algal symbiont for *Lophelia pertusa* and the lower temperature conditions found at coral reefs negates the first and mitigates the second of these hazards on the Norwegian margin [32]. Laboratory studies show that copious mucus release which may be triggered by *Lophelia pertusa* following particulate exposure [31, 33], coupled with the branched dendritic growth form of *Lophelia pertusa* further reduces (though not wholly removing) the danger of coral suffocation, even following environmentally high concentration, long term exposures [45, 31].

An overview of a typical Norwegian margin drilling event is given in Purser and Thomsen [22] but in summary, as a drilling is conducted, the broken up pieces of rock, the ‘drill cuttings cut from the well hole, are in the majority of cases released to the ocean. Such controlled releases of material is legal on the Norwegian margin, as drilling fluids (the material pumped alongside the drill bit to push waste material to the surface, maintain positive pressure and to cool the drill bit are commonly comprised of water and additives not considered environmentally toxic [46]. Fine particles of barite are often the primary additive in this process—the ‘weighting agent’. During some drill events, the rocks through which the drill passes may contain small quantities of oil originating from the target reservoir. Providing the content of the drill cuttings pushed out of the well hole remains at <1% oil, cuttings may be released to the ocean. If oil content exceeds 1%, then the cuttings must be shipped to shore for disposal. As a non-toxic, regulated material, release of drill cuttings to the environment cannot be compared to accidental releases to the environment, such as the petrochemical release (and subsequent flocculent deployments), occurring as a result of the Deep Water Horizon accident in the Gulf of Mexico, 2010 [47]. During production from a well, ‘produced waters’ may also be discharged to sea. These produced waters are not addressed in this study, though the interested reader is directed to the recent book edited by Lee and Neff [46] for an overview of the potential environmental impacts posed by this aspect of offshore production operation.

Drill cuttings can vary greatly in size and composition, in response to changes in the characteristics of the rock layers being drilled through, the speed of drilling and the composition of the drilling fluids [48, 49, 50]. All these variables may change on a fine temporal scale during drill operations [49]. Following discharge, the larger size fractions of cuttings form a cuttings pile close to point of release [29] whereas the finer material may be transported some distance before settling to the seabed [51, 25]. Predictive models of dispersion, such as the DREAM model [52, 28, 53] have been used operationally for a decade, with particulate size and density information feeding into an ocean current model driven by flow meter observations recorded from a drill location prior to drilling. Given that drilling operations commonly take ~a month and may take longer, depending on weather conditions and operational matters, oceanic flow conditions may differ from those predicted by the pre-drilling flow meter observations [30].

Larsson et al. [33] showed in the laboratory that dead coral skeleton and areas of *Lophelia pertusa* coenosarc in less than optimal health may become progressively smothered with fine drill cutting depositions over time. Under continual exposure to suspended drill cuttings at concentrations of 25 mg l^-1^ for 12 weeks, a clear visible accumulation of material on the coral structure can occur [33]. Additionally, settling material may remain on top of the less healthy coral polyps and branches after a single deposition event [31,32]. In the ocean, barite and barium have been found in the skeletons of *Lophelia pertusa* growing in close proximity to drill cutting releases during a drilling event in the early 1990’s [34]. At the time of the drilling described in Lepland and Mortensen [34] there was no knowledge that corals were present in the area, and therefore the concentrations of discharged material transported to the corals and the impact of any exposure on the coral reef communities remains uncertain. From the paper the impact on *Lophelia pertusa* was hypothesized to be minimal, with only very low concentrations of barite being found incorporated within living corals. There have been reports of corals growing on oil and gas production platforms [54], but these structures are put into position after drilling is completed and therefore the platforms, and colonizing corals, were not exposed to drill cuttings at time of initial drill cutting release. Possibly resuspended cuttings from the seafloor do indeed reach these corals in times of increased benthic flow, though this has not been investigated.

In this paper we present observations from a time series video monitoring campaign carried out at a number of small coral reefs within 2 km of a drilling site within the Morvin drill field on the Norwegian margin. By visiting reefs before, during, immediately after and a year after drilling the visual health status of the reefs were monitored over time. During the drilling process flow meters were deployed at several sites within a 2 km radius of the drilling rig to record local benthic flow dynamics. The data from these flow meters, in combination with information on the composition and volumes of material released to the ocean during the drilling process, were used in a transport model to predict the likely depths of material deposition on the seabed surrounding the drilling rig, and to produce estimates of likely suspended sediment concentrations reaching each of the monitored reefs throughout the drilling period. For a short period of several weeks, live video data were collected from a Lander deployed during the drill campaign—the results of this deployment are discussed in [55].

The overall aim of the study was to monitor and assess the behavior and abundance of *Lophelia pertusa* corals and reef communities at seven reefs in the vicinity of a drilling event, prior, during, immediately after and one year after drilling. Reefs selected were situated at various distances and in different directions from the point of drill cuttings release, and were therefore likely to be exposed to differing concentrations of drill cuttings (from no predicted exposure to occasional >25 ppm exposures). The key hypothesis under investigation was that the reefs exposed to the highest concentrations of suspended material would have a greater visual response evident than reefs exposed to lower concentrations, with these responses being changes in coral polyp activity, faunal community composition or increased sedimentation in the vicinity of the reefs.

## Methods

### 2.1 Location of drilling and monitored reefs

**Fig 1:**
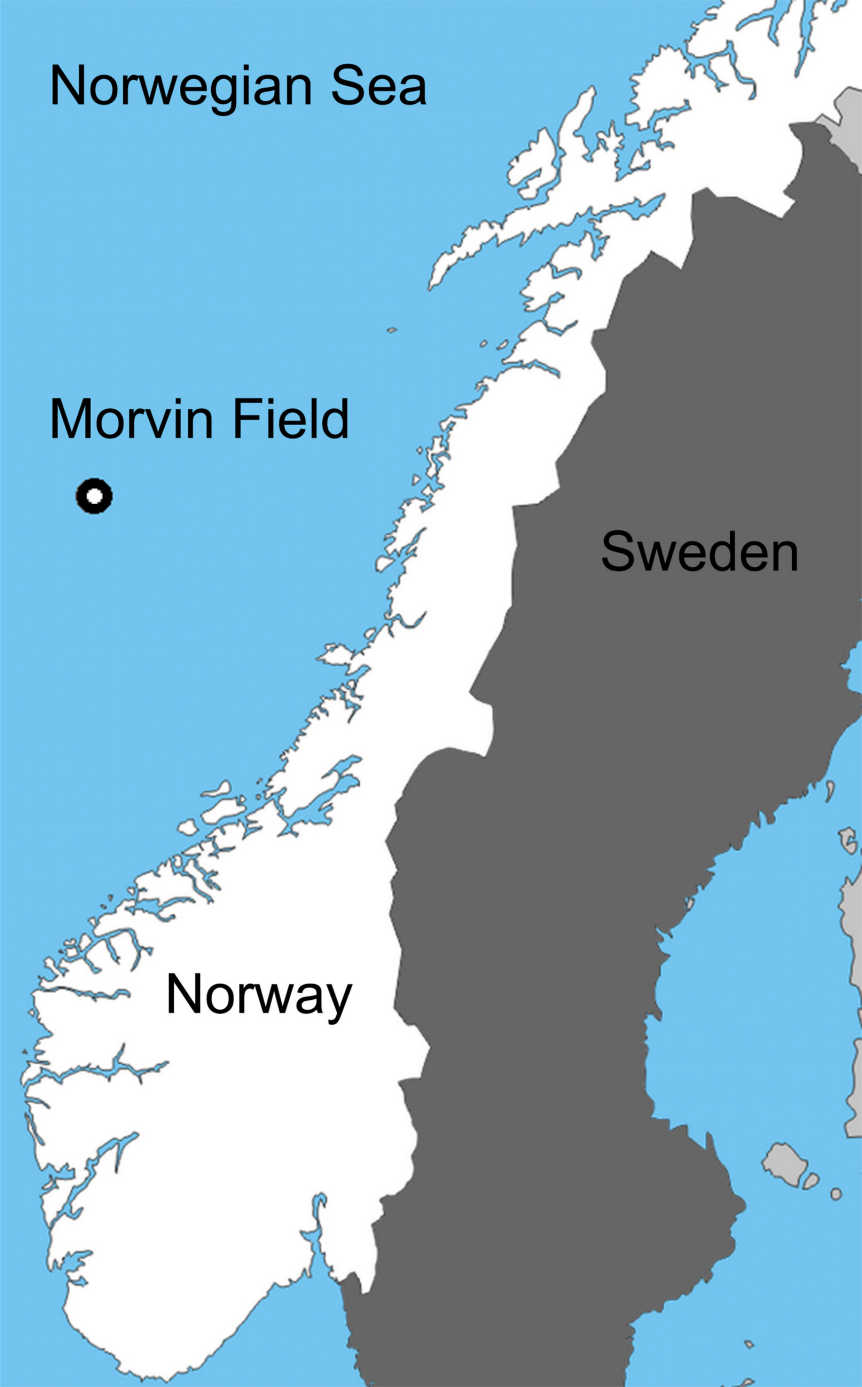
The location of the Morvin drill site on the Norwegian margin.

**Fig 2:**
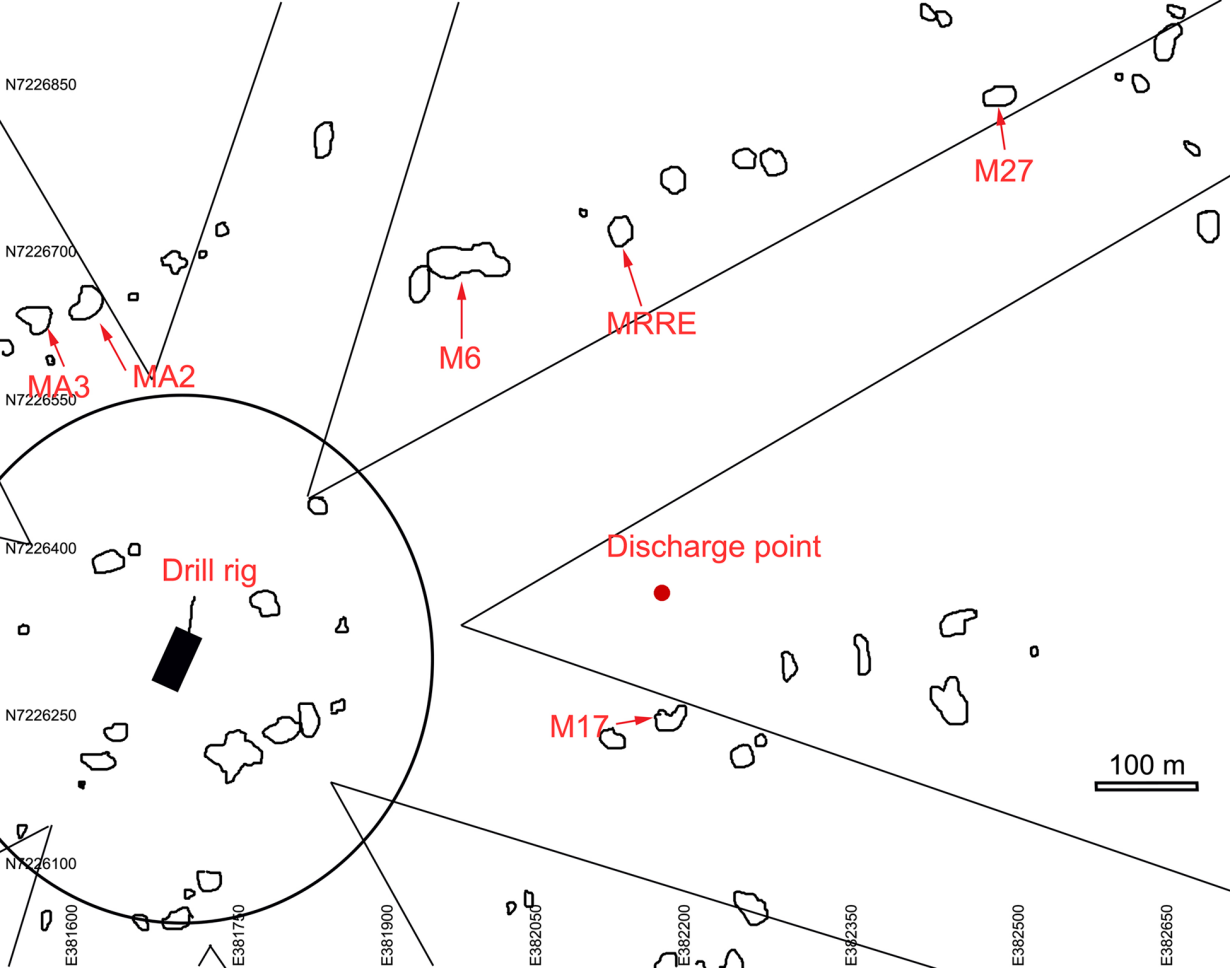
Map showing the location of the monitored reefs in relation to the site of drilling. Anchor corridors, seabed bathymetry and point of drill cutting release are also shown.

**Fig 3:**
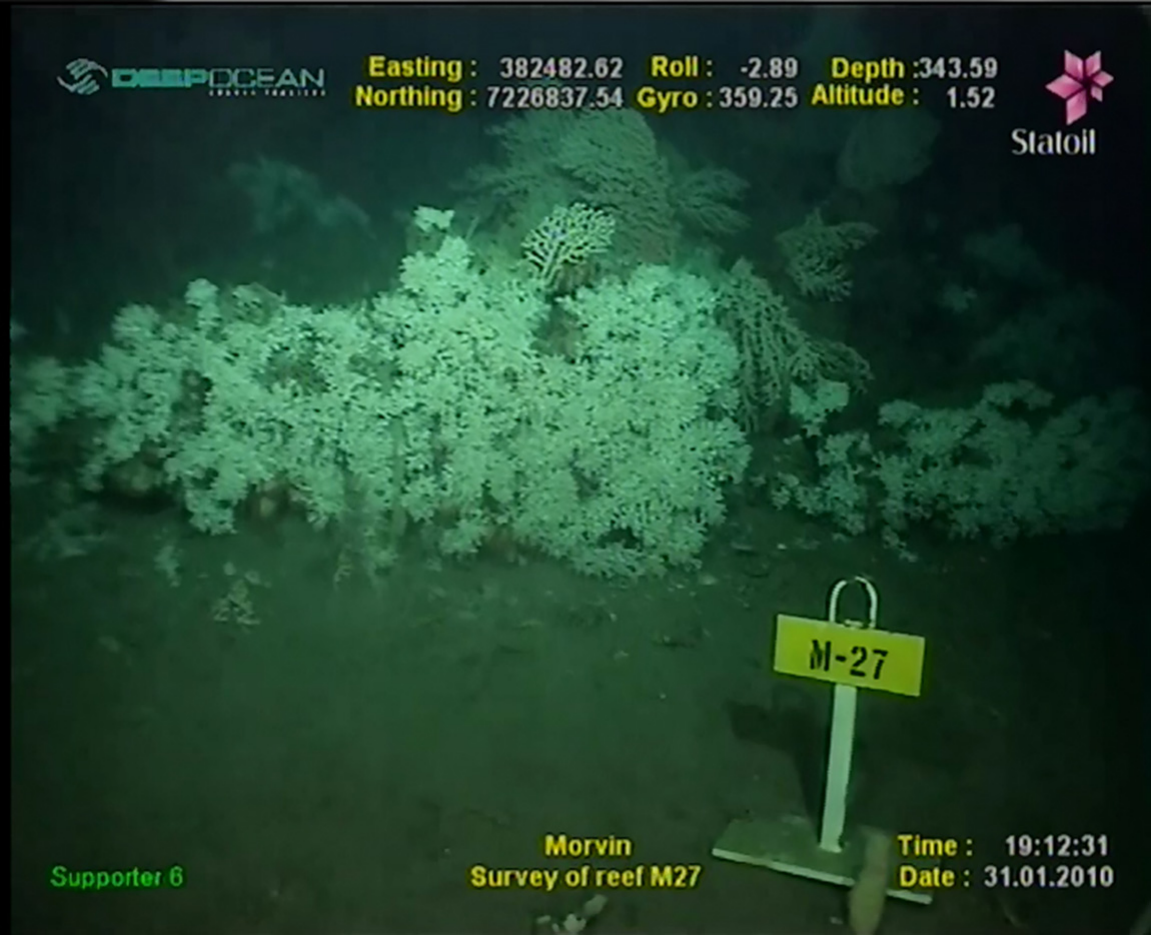
Typical reef marker. All reefs were marked with comparable markers, this is the marker positioned at reef M27.

With permission from the Norwegian Petroleum Directorate, four top hole sections and three full depth wells were drilled at the Morvin A location on the Norwegian continental margin between 9^th^ November 2009 and 9^th^ February 2010 (Fig 1). In advance of drilling, the MV *Skandi Bergen* installed the seabed infrastructure associated with drilling operations. Prior to this installation, the region was extensively mapped, with a number of coral reefs indicated by sidescan sonar survey and verified by ROV (Remote Operated Vehicle) dive survey [56, 57, 29]. These reefs were predominantly small in size, commonly less than 10 m in diameter with a maximum height above seafloor of <2 m. Of these verified reefs, Statoil selected those most suitable for time series study, selecting reefs located in various directions and at different distances from the point of drill cutting release (Fig 2). The main factors in deciding which reefs to focus studies on was to insure 1) reefs selected were situated in positions up and downstream of the cutting release point and 2) live coral be present on the reefs. A set of markers labeling each reef was installed by the MV *Skandi Bergen* ROV team (Fig 3). The reefs selected were similar in size to the living reefs of the Traena reef [13, 15, 34]; though at the Morvin location no evidence of the unidirectional growth observed at Traena reef was observed. These small reefs are also similar in size to those found within the Tisler reef province in the Skagerrak [10, 58], though more widely spaced from each other. The Morvin reefs are representative of many small reef regions on the Norwegian margin though dissimilar to the larger structures (with lengths of km) commonly found at the edge of the continental shelf in Norwegian waters [38].

### 2.2 Monitoring program

#### 2.2.1 Video data collection

Given that the position of the wells to be drilled was well known and constrained, and spatial arrangement of coral reefs in the 2 km surrounding the drill site mapped, the decision was made by Statoil to investigate and record with ROV mounted HD video a number of these small reefs, both before, during, immediately after and a year after drilling. The reefs in the drill area were primarily arrayed in two east north east-west south west chains, to the north and south of the drill location and point of cuttings release, with the closest being 100 m south of the point of drill cutting release (Fig 2). 9 reefs were selected for monitoring (Table 1), with initial video surveys of these carried out between the18^th^ October and 9^th^ November 2009, prior to drilling commencement (Table 2). The point of drill cutting release was selected to ensure that material would not be transported to the reefs in sufficient quantity to result in reef burial, thus following the ‘precautionary principle’. The immediate area surrounding a point of cuttings release is often wholly smothered by material, to a distance of tens of meters, depending on volumes of discharge [29]. From Fig 2 it can be seen that the selected reefs were arrayed around the point of drill cutting release and therefore each reef would likely be exposed to different concentrations of waste materials during drilling. A hydrodynamic survey conducted prior to drilling indicated the benthic flow regime to be stable at time of survey, with flow of bottom waters to be in a N / NW direction [30]. The predicted suspended particulate concentrations and deposited material thicknesses (see section 2.3) for each of the reefs to be monitored was modelled to remain below the potentially harmful concentration levels reported in [31, 32, 33].

**Table 1:** Co-ordinates of the reefs monitored during the monitoring campaign.

Reef code	Easting	Northing
M1	382127.3	7226710.1
MA1	381697.9	7226680.6
MA2	381608.7	7226634.7
MA3	381537.6	7226633.8
M6	381972.5	7226677.6
M17	382151.7	7226249.4
M17B	382113.9	7226218.5
M27	382484.0	7226834.0
MRRE	382122.7	7226709.4

**Table 2:** Dates on which each of the reefs in the monitoring campaign were visited, and usable video data of the reef collected.

Reef code	Before Drilling	During Drilling	After Drilling
M1	18/10/2009, 24/10/2009, 04/11/2009		
MA1	18/10/2009		
MA2	18/10/2009, 09/11/2009	14/11/2009, 16/11/2009, 18/11/2009, 22/11/2009	07/09/2012
MA3	24/10/2009		07/09/2012
M6	04/11/2009, 09/11/2009	14/11/2009, 16/11/2009, 18/11/2009, 22/11/2009, 28/11/2009, 02/12/2009, 03/12/2009, 04/12/2009, 10/12/2009, 11/12/2009	31/01/2010, 05/04/2011
M17	24/10/2009, 03/11/2009	12/11/2009, 13/11/2009, 15/11/2009, 16/11/2009, 17/11/2009, 18/11/2009, 20/11/2009, 22/11/2009, 27/11/2009, 28/11/2009, 29/11/2009, 30/11/2009, 03/12/2009, 04/12/2009, 07/12/2009, 09/12/2009, 10/12/2009, 11/12/2009	31/01/2010, 05/04/2011
M17B	09/11/2009	14/11/2009, 16/11/2009, 22/11/2009, 03/12/2009	31/01/2010
M27	04/11/2009, 09/11/2009	14/11/2009, 16/11/2009, 18/11/2009, 20/11/2009, 20/11/2009, 22/11/2009	31/01/2010, 05/04/2011
MRRE	04/11/2009, 09/11/2009	12/11/2009, 13/11/2009, 14/11/2009, 15/11/2009, 16/11/2009, 17/11/2009, 18/11/2009,19/11/2009, 20/11/2009,22/11/2009,02/12/2009, 03/12/2009, 04/12/2009, 07/12/2009, 10/12/2009, 11/12/2009	31/01/2010,05/04/2011

At each of the reefs selected for monitoring, site markers were installed to allow comparable video data to be collected on each survey visit (Fig 3). These repeat surveys were made throughout the first ~4 weeks of the drilling period (9^th^ Nov 2009—12^th^ Dec 2009). The numbers of visits to each of the individual reefs was not equal, with dive time being constrained by drilling operations and ROV availability. During these initial 4 weeks, the same ROV and camera setup was used, with a pair of forward facing 720 x 576 Kongsberg Colour Zoom (HD) cameras mounted, recording video at 720 x 576 pixel resolution. One camera was fixed in the forward position, with the second being manually controlled by the ROV team, and capable of taking close up images of polyp activity. In January 2010, April 2011 and September 2012 further visual inspections were carried out at the surveyed reefs. Dates on which each reef was surveyed is given in Table 2. During the January 2010 and September 2012 surveys, a fixed PAL 720 x 576 pixel ROV camera was used. For the April 2011 survey, two centre mounted Imenco zoom cameras were used in conjunction with VisualSoft encoding software to record digital (MPEG-2) video at 704 x 576 resolution. During these latter surveys, close up video was only collected occasionally.

From the full video dataset collected throughout the monitoring campaign, still images were extracted which allowed spatial comparisons of reef condition to be monitored over time. These still images were extracted from video frames which included the in-situ reef markers at each reef, and as much of the surrounding reef as possible (Fig 3 shows a typical example). These still images were labeled by reef, date and time of filming, then categorized as representing the reef as 1) Prior to drilling 2) During drilling 3) Immediately following the drilling campaign or 4) >1yr after drilling.

#### 2.2.2 Flow conditions, modelling of cutting dispersal and sediment trap deployments

Prior to drilling, current profilers were used to identify the prevalent current conditions throughout the area, a common procedure when planning a drilling campaign. These data were used operationally to predict the likely dispersal of released drill cuttings, using the DREAM dispersal model. During the drilling campaign, further flow meters were deployed. These flow meters were not equipped with live connectivity and could only supply data after the drilling event and instrument recovery, (thus precluding these data from being used in planning optimal times of drill cutting release to the environment). The results from these flow meter deployments are presented in Tenningen et al., [30]. The drilling campaign was initially envisaged to last approximately one month. In actuality, due to poor weather conditions and operational constraints this drilling period was extended into early February 2012. Drilling wells into the deep sea seafloor is not a continuous process, with operational considerations such as weather state, drill bit changing etc. resulting in periods of no drilling. The release to the ocean of drill cuttings is therefore not constant. The volumes and compositions of released materials throughout the drilling period are given in Table 3.

**Table 3:** Total quantities of material released during the drilling period.

Component	Total quantity released (tonnes)
Cuttings from bedrock	3296.63
Bentonite	537.32
Barite	934.97
Soda ash	6.20
Additional chemicals (predominantly cementing materials)	180.00
Water	91652.74
Total mud discharge	93092.00

### 2.3 Predictive modelling of drill cutting dispersal

Following drilling, the data recorded by the flow meters were used with the data on discharge volumes to provide a ‘hindcast’ report on the likely transport pathways of drill cuttings. The location and thicknesses of deposition layers across the drilling area and throughout the drilling period were modeled. Det Norske Veritas was contracted to produce these particulate transport concentration plots and distribution predictions by running their in-house predictive transport DREAM model. The modeled physical characteristics of the seafloor of the drill site were based on bathymetry data supplied by Statoil. Details on the composition of the drill cuttings (size characteristics, chemical information etc.) were also provided by Statoil, with volumes and times of drill cutting discharge supplied from the drilling platform. Hourly average estimated suspended particle concentration predictions were produced for each of the reefs surveyed and a final predicted drill cutting thickness deposition map produced for the areas surrounding the drill site. Both transport and deposition pathways were modelled by taking into account the average size and density ranges of drill cutting particles, with larger, denser drill cuttings settling more swiftly than finer or less dense material. Deposition depths were estimated by summing the concentrations of particles modeled to be deposited in each location at each time step of the model run.

To determine whether or not sediment could be observed in suspension at times of modelled sediment plume transport, visual assessment of suspended particle concentration was made from the reefs from 66 reef survey videos. These videos were selected as those containing periods with at least 5 minutes of reef footage where there was no seabed sediment resuspension resulting from ROV movement (i.e. sediment blasted into suspension by ROV thrusters). These videos were all recorded during the initial November—December 2009 drilling period.

**Fig 4:**
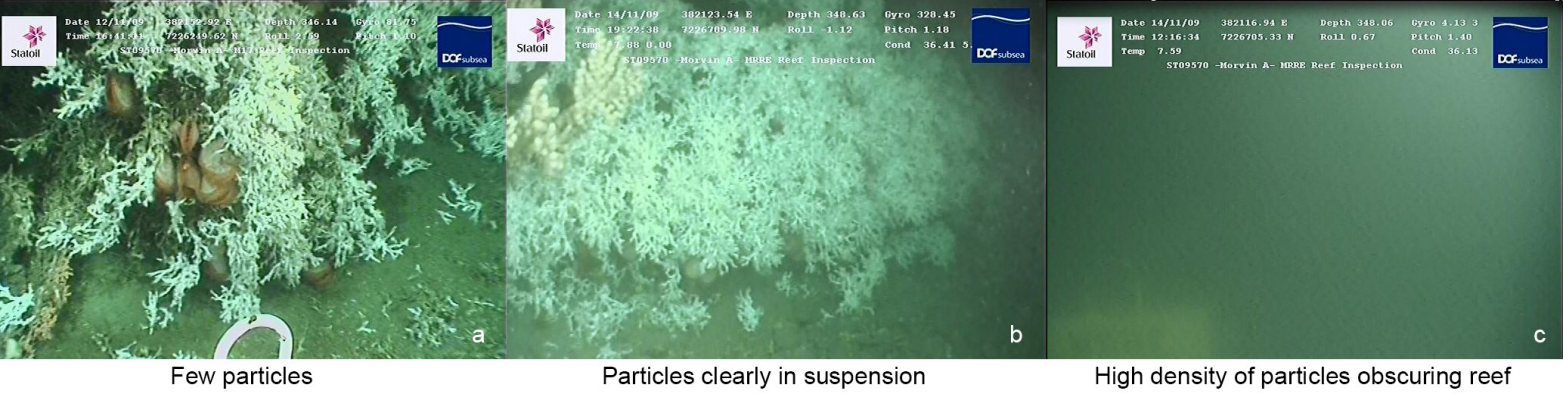
Examples of the three categories of visual suspended particle concentration used within the study. a) few particles visible, b) particles clearly in suspension, c) high density of particles obscuring reef.

The suspended particle concentration in each video was visually assessed as being in one of three categories, a) few suspended particles visible, b) suspended particles clearly visible and c) suspended particles in high enough density to obscure part of the reef (Fig 4). Following this visual assessment, the corresponding modeled concentrations were compared with this categorical data. The mean, median, maximum, minimum and mean standard deviations in modeled ppm drill cutting concentrations computed for each category of visual particulate concentration were also determined.

### 2.4 Responses to exposure

#### 2.4.1 *Lophelia pertusa* response to exposure

A video based approach was used to assess the health status of *Lophelia pertusa* at each reef. Direct methods of health status assessment, such as determination of coral living biomass, coral respiration rates etc. are not possible without direct sampling of the corals, and such an approach is not considered environmentally appropriate given the requirement for drilling campaigns to minimize impacts on reefs.

**Fig 5:**
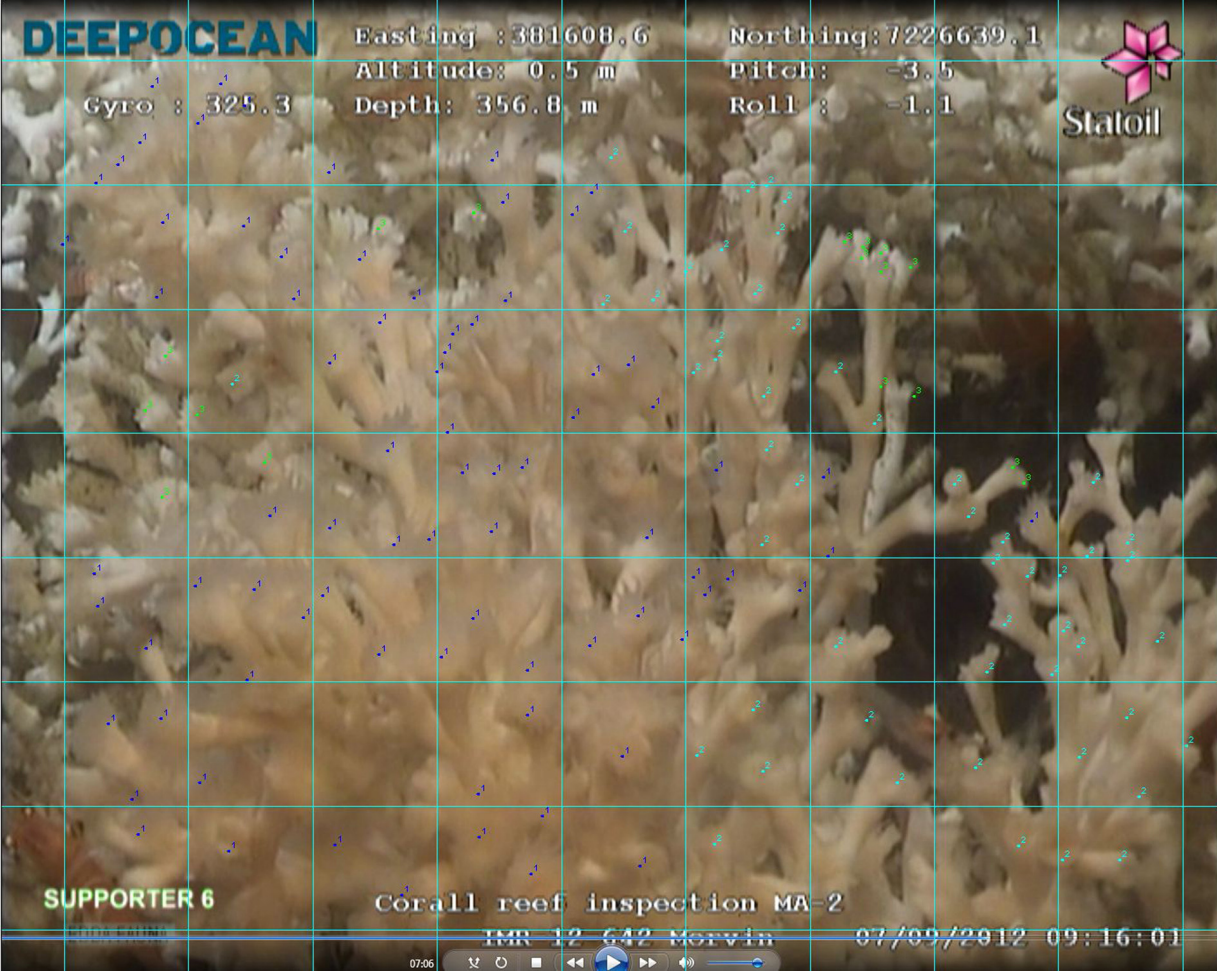
*Lophelia pertusa* tentacle extension state. 1) Dark blue numbered dots indicate fully extended tentacles, 2) Light blue numbered dots indicate visible, but not extended tentacles, 3) Green numbered dots indicate coral cups with no polyp tentacles visible.

*Close up video analysis of polyp behavior*. From video inspection, it is possible to assign a category to coral polyp behavior. Using the close up video data collected from the survey dives prior, during and at the end of the initial drilling period (Nov—Dec 2009) 4 still images were extracted from video collected at each of the surveyed reefs during each of these periods. These 4 images were selected to represent different areas of each reef, and were considered to be representative of the quality of video material available. Ideally more than 4 images would have been used in this analysis, but due to dive time constraints, particle resuspension from thrusters etc. 4 zoomed still images from each reef was the maximum number which could be extracted from all video material collected across all reefs. The polyps visible on each extracted image were logged using the ImageJ software [59] as being 1) extended 2) visible or 3) retracted, after Tenningen et al. [30] (Fig 5). As the surveyed reefs were arrayed in different directions and at various distances from the point of drill cutting release, it was likely (and predicted by the transport model) that the reefs were exposed to different concentrations of drill cuttings for different lengths of time. Following the output of the predictive transport model these reefs were categorically graded according to levels of likely exposure (Negligible, Occasional exposures >5 ppm, Repeated exposures <5 ppm). These data were then compared with an ANOVA test to determine whether or not there were clear behavioral differences between coral polyps located at reefs modeled to be exposed to different concentrations of particulates. A post-hoc Bonferroni test was used to determine between which levels (reef or observation period) any observed differences occurred. By also repeatedly observing each reef during each time period any change in behavior at a particular reef could also be assessed. For reefs modeled to be exposed to very low or negligible quantities of material, such observations would give an indication of the natural variability in polyp activity at a small reef in the area. For this analysis, the potential influence of different ROV thrusters, pilot operation techniques etc. must be considered as possible confounding factors, as well as additional, unmeasured environmental conditions at the times of observation (such as flow velocity, turbidity, suspended food availability etc.).

#### Change in reef form, colouration and associate megafauna abundance

Still images were extracted from the fixed camera collected video from each survey of each reef prior, during, at the end of drilling and >1yr after drilling. These extracted images were selected from the video stream to record comparable regions of each of the reefs, to allow change in the reef structure to be assessed over time. Six features were monitored throughout the images of each reef, wherever possible:

- Change in coral colouration
*Lophelia pertusa* rapidly changes from a bright white (or orange in some locations) to a dull brown/orange following polyp death. In this study the regions of live coral visible from the pre-drilling images were compared with those after drilling, and a qualitative assessment of ‘no color change’ or ‘colour change’ given for each image taken after commencement of drilling. Though different camera setups on the various ROVs captured colour in different hues, these differences were assumed to be insufficiently pronounced to prevent differentiation between living tissue and brown dead coral skeleton.
- Sediment accumulation within coral structure
  The amount of sedimentation visible within the coral structure within each image was qualitatively assessed as ‘no sediment’, ‘some sediment accumulation visible’ or ‘heavy sediment accumulation visible’.
- Sediment accumulation at reef base
  The amount of sedimentation visible at the base of the coral structure within each image was qualitatively assessed as ‘no increase in sediment’, ‘some sedimentation increase’ or ‘heavy sediment accumulation’.
- Loss of gorgonian corals
  The diversity of CWC reef fauna on the local scale is well reported [60, 61, 62, 15]. Within the vicinity of some of the reef markers deployed in this study gorgonian corals *Paragorgia arborea* and *Primnoa resedaeformis* were visible in the pre-drilling extracted images. As relatively slow growing species [63, 64, 65] it is unlikely that any new gorgonian colonies would establish themselves and reach an observable size within the survey period, so gorgonian abundance was qualitatively assessed as ‘no change’ or ‘gorgonian decline’ throughout the survey images.
- Loss of *Acesta Excavata*
  As with the gorgonian corals, the large bivalve *Acesta excavata* species was visible, sometimes in abundance, within some of the pre-drilling images. Shell abundance was qualitatively assessed as ‘no change’ or ‘decline’ throughout the survey images.
- Increase in coral fragment abundance
  Following reef damage or polyp death, bioerosion may be swift in *Lophelia pertusa* colonies [66, 67] with such erosion leading to an increase in fragment abundance at the bottom of reef structures. In regions with periodic high flow velocities these fragments may be dispersed over time. For this study, the following categories of qualitative assessment were assigned: ‘Initial fragments only’, ‘Some new fragments’, ‘Many new fragments’ or ‘Fragments buried or disbursed’.

A more quantitative assessment of reef structure change over time was not possible, given that the ROV pilots did not position the vehicle in exactly the same place during each survey dive, and that different camera rigs were used at each time step.

## Results

### 3.1 Modelled dispersal predictions

In situ flow data measurements, combined with the times and compositional details of drill cutting release to the ocean (total quantities given in Table 3) allowed concentrations of material in suspension within the lowest few meters of the water column to be estimated for any hourly period within the 125 day monitoring period, across the Morvin region. Unfortunately, there was no in situ flow meter deployed and functioning correctly for the period of drilling in January and February 2010. To allow transport within this period to be modeled, the average flow conditions measured in November and December 2009 were assumed to be prevalent during January and February 2010; therefore results for this period should be considered with caution.

**Fig 6:**
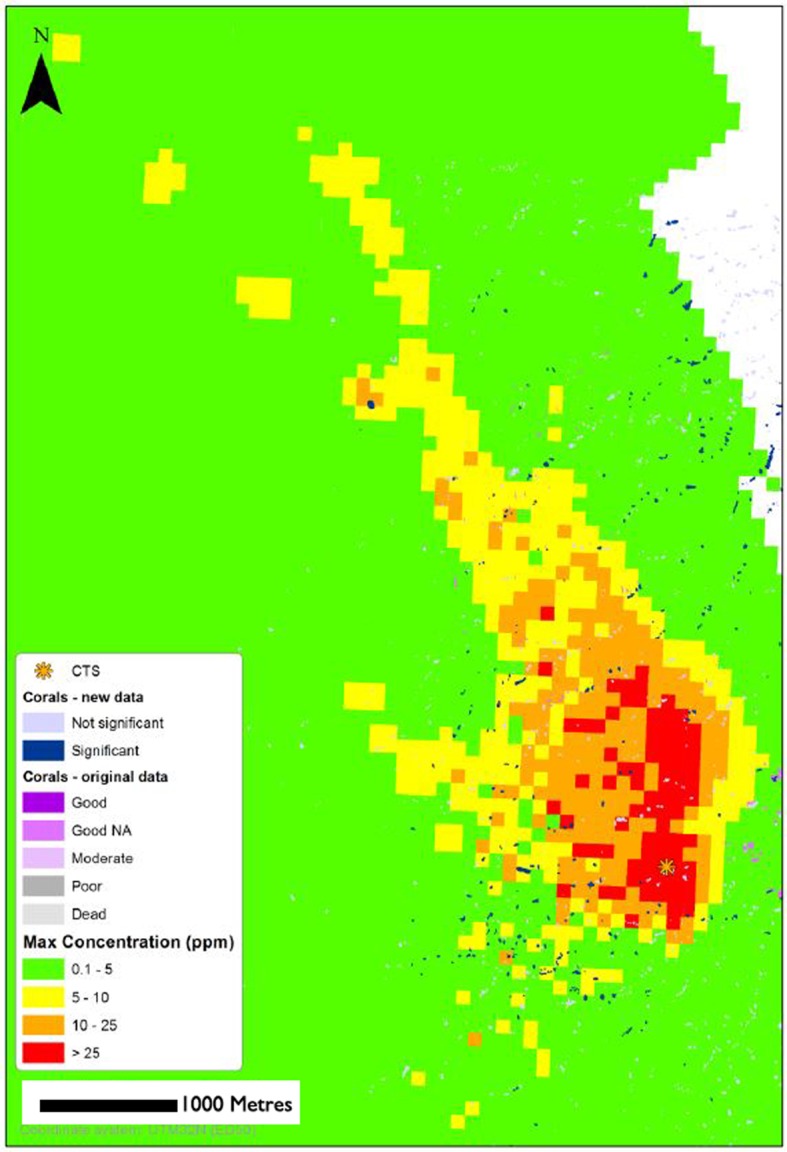
Modelled suspended particle concentrations within the vicinity of drilling during the monitoring period. ‘CTS’ represents the point at which drill cuttings were released to the ocean. The ‘Corals-New data’ are coral reefs visited by ROV within the year prior to drilling, whereas ‘Corals-original data’ are previously reported reefs. ‘Max concentration’ is the maximum concentrations of suspended drill cutting particles modelled to be transported to each grid square at some point during the drilling period.

Presenting the whole hourly output of the modellng endeavor within the context of this paper is not possible. Fig 6 gives a suspended particle concentration plot for a one hour period following one of the highest volumes of drill cutting release. As can be seen in the Fig, concentrations in excess of 25 ppm (concentrations which may impact negatively on *Lophelia pertusa* growth if maintained for periods of >12 weeks [31, 33] extend several hundred meters north of the point of cuttings release, and also some 100 m south of point of release, encompassing several of the monitored reefs. Lower concentrations of particles are extended in a plume running roughly NNW—SSE.

**Fig 7:**
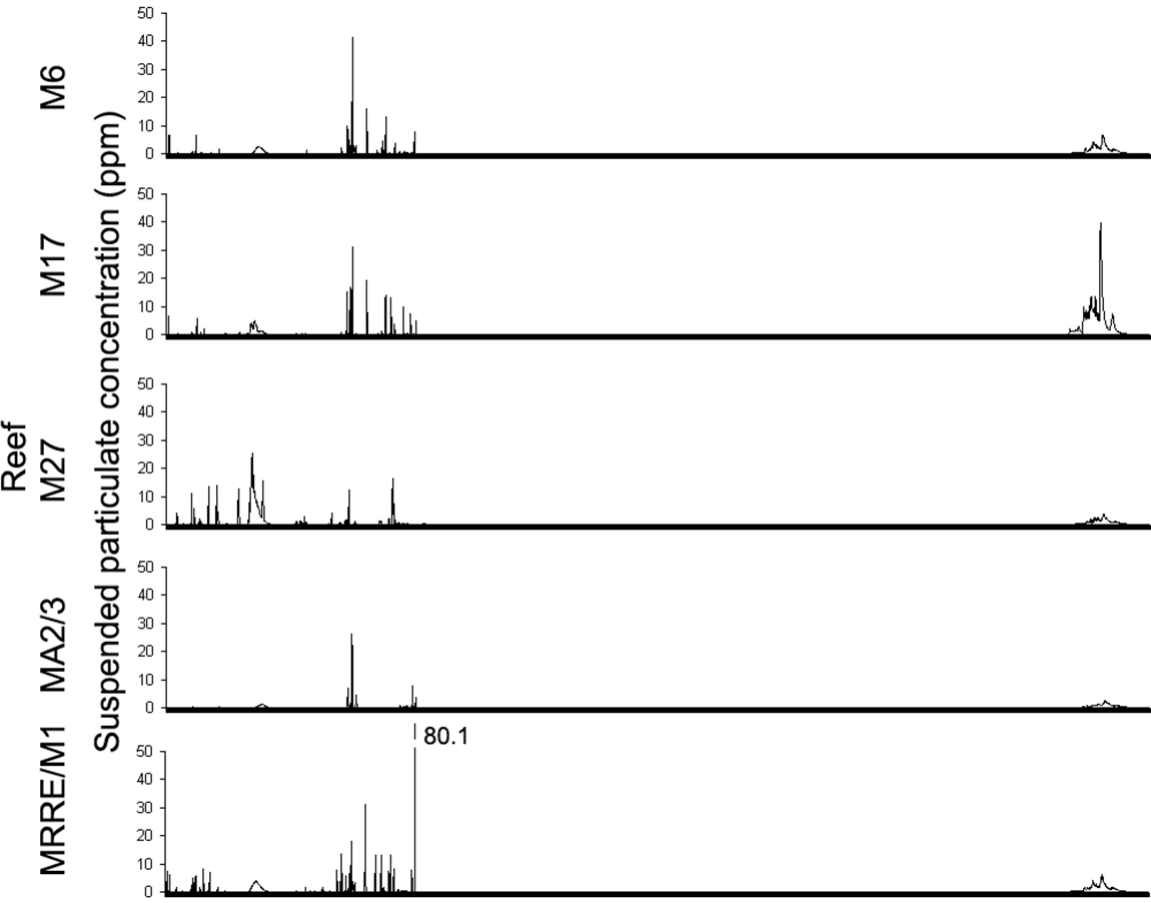
Modelled particle concentrations at each surveyed reef throughout the monitoring period. Data shown is average hourly concentrations of material modelled to be present in suspension at each reef during the monitoring period.

Fig 7 gives the modeled particulate concentrations predicted by the model to be found at each investigated reef throughout the 125 day drilling period. Prior to drilling, advance flow velocity measurements had indicated that the reefs labeled M27 and M17 would likely be not exposed to drill cuttings, with all discharged material being carried in other directions. As can be seen from Fig 7, flow velocity measurements taken during the drilling period indicate that this was unlikely to have been the case. These two reefs, along with reef MRRE likely experienced a greater number of periodic plumes of material of >25 ppm concentration during the drilling events than the other monitored reefs. MA2 and MA3 were likely exposed to the lowest volumes of drill cuttings.

From the modelling results it appears unlikely that any of the monitored reefs was exposed to concentrations of drill cuttings in suspension of >25 ppm for more than a few days in total.

Modelled depositional thicknesses of released drill cutting material resulting from the whole drilling period were generally higher close to the point of drill cutting release. In Table 4 the modeled estimations of material deposition thicknesses are given for each of the monitored reefs, with both the modeled thicknesses for the November—December 2009 period (that with accurate flow data available) and for depths which also include the January-February 2010 period (for which accurate flow data was not unavailable). Table 4 shows drill cutting depositional thicknesses of between 1.5 and 2 mm to be likely to occur at the M17, MRRE and M1 reefs, with less material settling from suspension at any of the other monitored reefs.

**Table 4:** Modelled total thickness of depositional drill cutting layer deposited at each of the monitored reefs. Table gives figures incorporating both the likely depths resulting from the November—December 2009 drilling period (where accurate flow data was available) and depths with the estimated January-February 2010 drilling period added (during which no flow data was available).

Reef	Modelled sediment thickness (Dec 2009)	Modelled sediment thickness (Feb 2010)
M6	0.04 mm	0.05 mm
M17	0.7 mm	0.82 mm
MRRE	1.2 mm	0.64 mm
M1	1.2 mm	0.64 mm
M27	0.17 mm	0.25 mm
MA2	<0.001 mm	0.001 mm
MA3	<0.001 mm	0.001 mm

### 3.2 Modelled suspended sediment concentrations and visual assessment of particulate concentration

**Fig 8:**
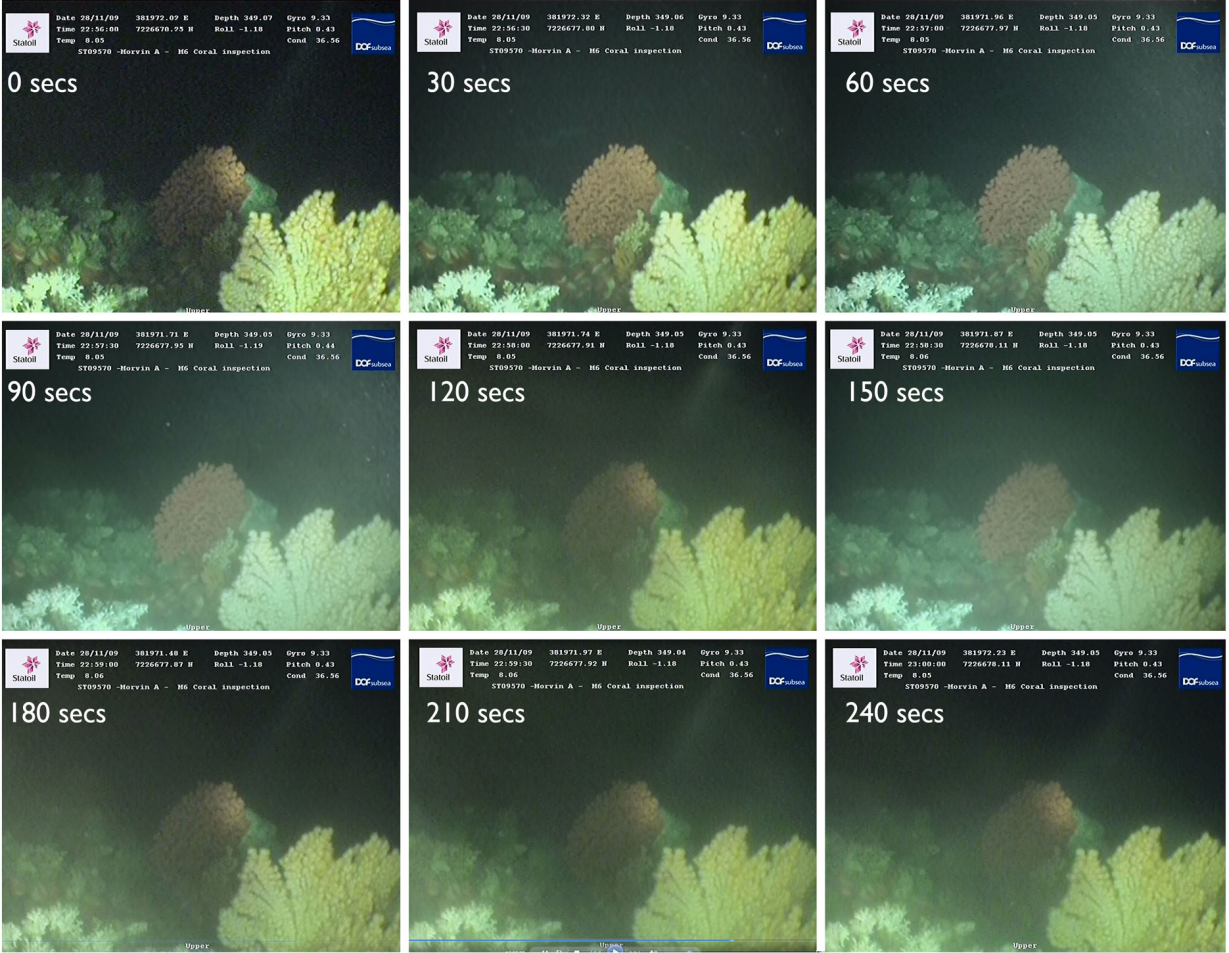
Change in suspended particle concentrations at monitored reefs over short timescalesduring a drilling event. Here time series images taken with a landed ROV show concentrations of drill cuttings in suspension increasing from few or no particles in suspension to a density sufficient to partially obscure the over a four minute period. The ROV recording these images had landed prior to the commencement of image capture, and therefore turbulence from the thrusters is not the cause of the suspended particle concentration observed.

Increases in mean average modeled ppm concentrations generally correlated with increase in visual particle assessment category (Table 5). This modeled correlation was not exact however, as the sizable standard deviations in predicted concentrations attests. It is interesting to observe that although only 5 dives were categorized as occurring in waters with sufficient densities of particles to obscure the reef, the hours during which each of these dives took place were modeled to contain elevated concentrations of particles in suspension (see minimum predicted concentration, Table 5). There were high peaks of >15 ppm predicted during some of the dive hours in which ‘few particles’ or ‘clear suspended particles’ were manually recorded. These high peaks were clearly not recorded during the dive videos which were often of only a few minutes duration. In Fig 8 it can be seen how swiftly the visual concentration of particles in the water column above the reef can change. Over a 4.5 minute period, all three categories of visual particle concentration are apparent. The images making up this image sequence were taken from a stationary ROV positioned at time of drill cutting release to specifically observe for any visual evidence of plume arrival at the reef. The 4.5 minutes of footage were extracted from a longer video sequence (S1 File) showing that the plume passage across the reef was swift.

**Table 5:** Modelled suspended concentrations of drill cuttings at times of visual particle concentration assessment. Three grades of particle concentration were used: 1) no or few particles, 2) particles clearly in suspension, 3) reef partially obscured by high particle densities. The mean, median, maximum, minimum and mean standard deviation in modelled values corresponding to each of these three visual categories is given.

Modelled parameter	No or few particles	Particles clearly in suspension	Reef partially obscured by particles
Number of visual observations	41	20	5
Mean modelled concentration (ppm)	0.78	0.88	1.44
Standard deviation in mean (ppm)	2.39	3.78	1.57
Median modelled concentration (ppm)	0.00	0.00	1.36
Maximum modelled concentration(ppm)	12.94	16.93	3.85
Minimum modelled concentration (ppm)	0.00	0.00	0.03

### 3.3 Response of *Lophelia pertusa* polyps to drill cutting exposure

The monitored reefs were exposed to different quantities of drill cuttings throughout the drilling period (Fig 7). Prevalent current conditions likely exposed reefs MA2 and MA3 to less suspended material than the other monitored reefs, with Table 4 indicating that quantities of settling material at these two reefs to have likely been several orders of magnitude less than at the other reefs—negligible quantities of material. Table 6 gives the percentages of polyp tentacles expanded, partially expanded or retracted prior, during, at the end of and after drilling for each reef where such polyp monitoring was carried out (Fig 4 indicates how these three states differ visually). Given operational constraints, camera suitability and time availability of ROVs, there are some sampling gaps in this table. The percentages of fully extended polyps observed at each of the surveyed reefs and reported in Table 6 were compared with an ANOVA test. Few significant differences in percentages of fully extended polyps were indicated by the ANOVA test (Table 7). When considering this analysis it is important to consider that there was a discrepancy in the number of reefs assessed before, during at the end of and after drilling for this test, with prior and post drilling analyses being particularly sparse. These differences were indicated by the Bonferroni post-hoc test to be between observations of the M6 reef during drilling and the M17 reef, prior and during drilling. As Table 6 shows, M6 and M17 were not modeled to be exposed to very different concentrations of material. The high standard deviations in the percentages of polyps exhibiting each behavioral type observed at each reef during each time period (pre drilling, during drilling etc.) indicate a great variability in polyp behavior across each reef. Fig 5 is a typical example of the kind of close up image extracted from the collected video data—the polyps in the image exhibiting the various behaviors are not randomly intermixed, but clustered together into rough groups (in Fig 5, the majority of extended polyps are on the left of the image, the visible polyps to the right). Video data varied significantly in quantity, stability, focal length, duration and illumination throughout the study, dependent on which ROV was deployed and pilot strategy. This variability is to be borne in mind when considering the presented results. A key observation made at all reefs surveyed was that many polyps were clearly alive and active at all times of observation, regardless of modeled particle exposure conditions.

**Table 6:** Percentages of polyps Expanded, Partially expanded or retracted at each of the survey reefs prior, during and after the drilling campaign.

Reef and modelled exposure level	Polyp behaviour	Prior to drilling	During drilling	End of December drilling	Post drilling
MRRE (regularly exposed)	Expanded		32.6 (+/-11.1)	53.6 (+/-8.8)	
	Partially expanded		52.9 (+/-9.0)	40.6 (+/-9.7)	
	Retracted		14.5 (+/-3.6)	5.8 (+/-4.0)	
M17 (regularly exposed)	Expanded	26.6 (+/-15.0)	21.2 (+/-6.4)	32.6 (+/-19.0)	
	Partially expanded	29.9 (+/-5.2)	55.1 (+/-8.5)	34.6 (+/-4.5)	
	Retracted	43.6 (+/-12.4)	11.8 (+/-7.1)	32.8 (+/-20.9)	
M6 (regularly exposed)	Expanded		70.9 (+/-16.4)	34.6 (+/-24.0)	
	Partially expanded		22.4 (+/-10.0)	51.0 (+/-21.7)	
	Retracted		6.7 (+/-6.6)	14.3 (+/-5.9)	
M27 (Periodically exposed)	Expanded		39.9 (+/-9.2)	31.8 (+/-20.4)	
	Partially expanded		48.4 (+/-12.7)	43.8 (+/-14.6)	
	Retracted		11.7 (+/-7.1)	24.3 (+/-7.3)	
MA2 (Low occasional exposure)	Expanded		61.8 (+/-10.6)		30.0 (+/-30.2)
	Partially expanded		25.7 (+/-9.8)		52.3 (+/-23.0)
	Retracted		12.5 (+/-2.7)		17.7 (+/-15.2)

+/- indicates SD.

**Table 7:** ANOVA output of one-way test to assess whether or not percentages of fully extended polyps differed by reef or period of observation.

	Sum of squares	Df	Mean square	F	Significance
**Between reefs**	9810.547	10	981.055	3.37	0.004
**Within period of observation**	9597.888	33	290.845		
**Total**	19408.435	43			

All observations from Table 6 analysed in the ANOVA.

### 3.4 Change in reef form, colouration and associate megafauna abundance following exposure

As described in section 2.2.1 different ROVs, lighting, camera rigs, vessels and ROV pilots were used throughout the monitoring period. This resulted in the video data collected throughout the campaign being variable in resolution, illumination and stability. In some instances ROV pilots landed ROVs within close proximity of monitored reefs, in others ROVs were hovered at a distance from the reef. Depending on ROV design, this hovering had a variable effect on image quality by resuspending differing amounts of material from the seafloor. This variation in ROV piloting and image collection methodology was a major drawback with the monitoring program presented here, and in future monitoring events the dive protocol used should be fixed across all dives.

**Fig 9:**
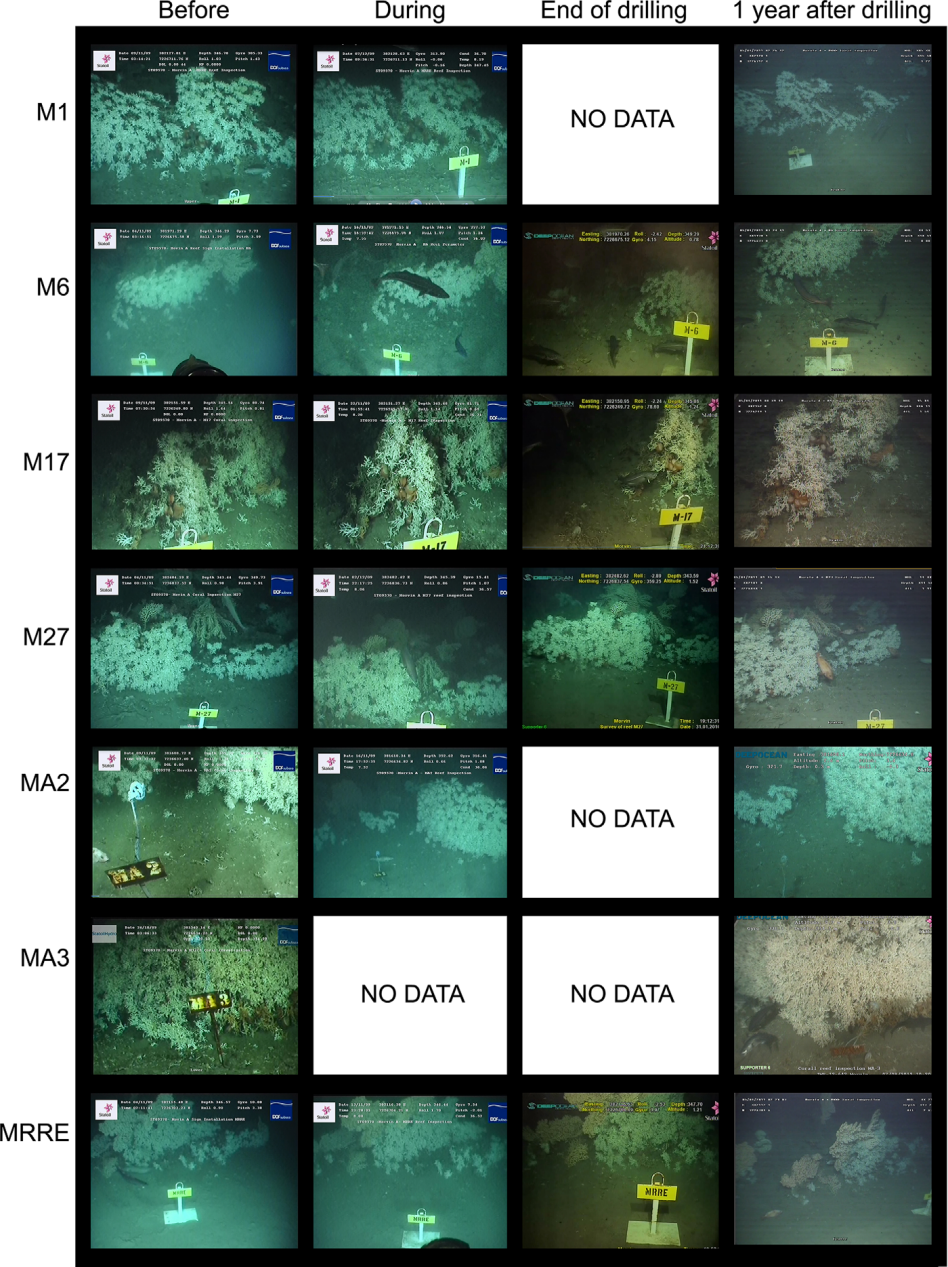
Time series images taken of each of the monitored reefs throughout the monitoring campaign. 1) prior to drilling, 2) during drilling, 3) Immediately following the drilling period and 4) >1yr after drilling.

Video image grabs from each of the monitored reefs representing each reef 1) prior to drilling, 2) during drilling, 3) Immediately following the drilling period and 4) >1yr after drilling are given in Fig 9.

Table 8 gives a summary of the qualitative visual observations made of sediment deposition, *Lophelia pertusa* fragment, gorgonian coral and *Acesta excavata* abundance over the monitoring period. Whenever observed, *Acesta excavata* shells were generally open, regardless of suspended particulate presence / absence (data not shown). Where gorgonian corals were observed, and observed repeatedly on follow up visits, clearly the same coral colonies were present, and in comparable health (i.e. no dead branches, standing vertically in the water column). To summarise the table, there was no apparent degradation of overall *Lophelia pertusa* reef health apparent at any reef during the monitoring period, regardless of level of suspended or settling drill cutting exposure. The only clear changes over time in reef condition apparent from the collected images was an increase in observable small coral fragments in the vicinity of many reefs during and after the drilling period. Such an increase in small fragments was not observed at the least exposed monitored reef (MA2) but there were indications of change in visible fragment abundance at all the other reefs.

**Table 8:** Qualitiative assessment of the change in reef appearance over time. Where no suitable reef images covering a comparable area of reef was available, ‘NO DATA’ has been entered. Where the viewing angle is inappropriate for a particular observation to be made ‘UNSUITABLE IMAGE’ has been entered.

Observational parameter	Reef	During drilling	End of drilling	>1 year after drilling
**Colour Change**	All surveyed reefs	No change	No change	No change
**Sediment accumulation within coral structure**	All surveyed reefs	None visible	None visible	None visible
**Sediment accumulation surrounding base of reef**	M1	Some accumulation visible	NO DATA	No accumulation visible
	M6	No accumulation visible	Some accumulation visible	No accumulation visible
	M17	No accumulation visible	No accumulation visible	No accumulation visible
	M27	No accumulation visible	No accumulation visible	No accumulation visible
	MA2	No accumulation visible	NO DATA	No accumulation visible
	MA3	NO DATA	NO DATA	No accumulation visible
	MRRE	Some accumulation visible	Some accumulation visible	Some accumulation visible
**Loss of gorgonian corals**	M17	No loss	NO DATA	No loss
	M27	No loss	No loss	No loss
	MRRE	No loss	No loss	No loss
**Loss of *Acesta excavata*?**	M1	No loss	NO DATA	No loss
	M17	No loss	No loss	No loss
	M27	No loss	No loss	No loss
	MA3	NO DATA	NO DATA	No loss
**Increase/decrease in coral fragment abundance**	M1	Some new fragments	NO DATA	UNSUITABLE IMAGE
	M6	UNSUITABLE IMAGE	UNSUITABLE IMAGE	UNSUITABLE IMAGE
	M17	Some new fragments	Some new fragments	Some new fragments
	M27	UNSUITABLE IMAGE	Fragments buried or disbursed	Some new fragments
	MA2	Initial fragments only	NO DATA	Initial fragments only
	MA3	NO DATA	NO DATA	UNSUITABLE IMAGE
	MRRE	Fragments buried or disbursed	Fragments buried or disbursed	Initial fragments only

## Discussion

Drill cuttings discharged to the ocean during the Morvin A drill campaign discussed in the current study contained only water based drilling muds. These cuttings therefore consisted exclusively of the drilling muds, the bedrock through which the drill cut, a total of <1% hydrocarbons (with any hydrocarbons present originating within the rock layers above the reservoir rocks) and low concentrations of a few non-toxic additives, mostly cementing material to firm up the drill holes (Table 3). Discharge of drill cuttings cut with oil based muds, or of cuttings originating from different lithologies than those drilled through at the Morvin field in this instance, may have a different impact on *Lophelia pertusa* and / or associate reef fauna than those reported here. Although in most European waters oil based muds and cuttings are no longer discharged to the ocean, historically the practice did occur, and elsewhere in the world oceans is ongoing. Oil and water based drill cuttings have been shown to have very different impacts on benthic communities outside of the reef environment, with the long-term effects of oil based cuttings the more severe on benthic communities [68]. Throughout this section it is to be borne in mind that water based drill cuttings are the focus of discussion only.

### 4.1 Visual impacts on *Lophelia pertusa* behavior during and after drill cutting exposure

Polyp behavioral change during and following environmental stress has been reported in cnidarians [69] and corals in particular [70, 31, 33]. With CWCs, the validity of identifying tentacle retraction as a response to environmental stress has been difficult to establish unambiguously. In the laboratory, freshly collected *Lophelia pertusa* corals will retract their polyp tentacles in response to vibrations and handling (personal observation) and in response to the delivery of individual pulses of fine grained settling material [31]. Such responses seem to be short lived, with polyp behavior reverting to pre-exposure levels within a few hours [33]. During extended exposures to suspensions of material (12 weeks constant exposure) to concentrations of suspended drill cuttings or resuspended seafloor sediments at concentrations of <5 mg l^-1^, polyps of *L*. *pertusa* seem to adapt to the new prevalent environmental conditions with similar percentages of polyps remaining extended as observed pre-exposure. However, during exposures of the same duration but to concentrations of 25 mg l^-1^ the percentage of extended polyps was observed to be reduced by ~50% [33]. Polyps wholly buried under sediment will no longer extend their tentacles and die within a few days of being covered [71, 32], but given the structural form of *L*. *pertusa*, burial of live polyps is unlikely in the environment, with settling material readily falling from coral branches [31] possibly assisted by mucus release [32] and height above the seafloor of the living polyps.

Outside of the laboratory, the current study is the only one to date (of which we are aware) that has attempted to monitor polyp behavior throughout the whole of a drilling campaign. Unfortunately, the attempt to monitor visually in HD from a static lander in real-time the behavior of corals failed for technical reasons early during drilling [30] rendering the data presented here from the ROV dives the most complete temporal set thus far collected. By using the various ROVs employed in the monitoring campaign, reefs exposed to a number of predicted concentration pulses of >25 ppm were repeatedly visited and polyps filmed in close up (MRRE, M17), as were reefs exposed to fewer pulses of such concentration (M27] or concentrations of ~ = <10 ppm (M6) and those modeled to have never been exposed to concentrations of cuttings greater than a few ppm during the drilling campaign (MA2). In all cases, the high standard deviations in polyp behavior counts varied on a comparable scale across individual reefs as between reefs, whatever the modeled exposure conditions, indicate that at the levels and durations of particle exposure experienced by the reefs during this study, no behavioral change was triggered in *Lophelia pertusa*. Percentages of corals observed to be fully extended in the current study were ~20% less than those reported in unexposed corals in the laboratory [33].

The reasons for the great variability in polyp behaviour across individual reefs are likely numerous. The complex relief structure of *Lophelia pertusa* reefs has been shown to result in highly heterogeneous flow conditions on a local scale [72] resulting in suspended food recirculation in some areas, or flow velocities particularly favourable for food capture by polyps in others [73, 10, 74]. Possibly the action of ROV thrusters and the associated resuspension of seafloor particulates played a role in determining coral behaviour. As outlined in 2.2.1 and 3.3, ROV piloting techniques varied greatly with dive. These noisy vehicles can create many vibrations, as well as directed thrusts of water, which may influence polyp behaviour. Certainly the patchiness of behaviour is not solely the result of ROV activity, with fixed cameras observing such patterns in unexposed reef environments [58] and during the first weeks of drilling activity during this Morvin A monitoring campaign [30]. All of these confounding factors add up to the likelihood of polyp activity only being possibly useful as a crude measure of response to major disturbance in the field, and as a guide for determining whether or not *L*. *pertusa* is stressed by an exposure event, is likely an approach of little practical application. Possibly, the number of close up images of areas of reef taken during each time period of the current study was insufficient (4 close up images of polyp activity from each reef at each time stage), though variability has also been apparent in analyses of larger data sets [30]. A clear observation from the data collected here is that despite the various shortcomings of the monitoring techniques, live corals are present at each of the reefs during and after drilling (at least at times of observation) and drilling did not lead to any mass fatalities of corals.

### 4.2 *Lophelia pertusa* reef condition, during and after drill cutting exposure

#### Absence of visible sedimentation

From ROV video data (both zoomed video recording on the scale of individual polyps and data collected covering the larger reef structure) it appears that from the concentrations of suspended material released during this drilling campaign, no visible sediment accumulation was apparent within or surrounding *Lophelia pertusa* reefs at any time during the monitoring campaign at any of the monitored reefs. Although close up video and / or still images were not collected from the seabed around the reef structures, the colouration of the seafloor was similar before, during and after drilling, likely indicating no significant coverage by drill cuttings. In Gates and Jones [29] they report visible seabed deposition of drill cuttings around a comparable drill well to extend up to a few hundred meters from point of release. The majority of the reefs monitored in the current study were at distances of approximately 200 m or greater from point of release, with the exception of M17, 150 m south (and in the predicted upstream direction) of point of release. Though drill cutting concentration exposures at each monitored reef was predicted from modelled data, it can be assumed that given the position and distance of the various reefs around the point of drill cutting release exposure would likely have occurred at some of the reefs at a minimum level, even if modelling was not particularly accurate. Variations in size composition of waste material [50] and hydrodynamic behavior of drill cuttings also confound modelling accuracy [49]. It is likely that some amount of drill cuttings entered between the polyp branches to within the reef structures, unobservable by ROV mounted video cameras. The physical complexity of these reefs offer habitat for small mobile fauna such as crustaceans within the coral branches [75], the microhabitats within the branches experiencing lower flow velocities than found at the outer fringes of a coral colony. It is likely that the higher flow conditions in the fringe areas, coupled with coral mucus release, ensure the cleanliness of these reef sub-habitat regions—the regions visible to ROV camera rigs. Within the reef branched structure, transported material would likely drop from suspension onto coral polyps, and possibly remain there, entrapped within exuded mucus but not slipping from the coral branches due to the high density of coral structure. In the natural situation, this variability in flow velocity across the interior of a colony, and the infilling of the central region by sediment falling from suspension, has been proposed as the main cause of reef growth for some reefs [76]. In reefs previously exposed to drilling, drill cuttings have been found within coral skeleton [37]. During the 1992 drilling event described in Lepland and Mortensen [34], it was unknown that corals were situated 500 m downstream from point of cuttings release, though in the absence of both accurate flow and drill cutting volume and composition discharge data being available for both drilling events (The 1992 event and the current study), it is difficult to accurately compare the two events. It would seem likely however that some amount of cuttings reaching reefs MRRE, M1, M17 etc. will have been incorporated into the skeletons of live corals.

To accurately gauge the amount of drill cuttings reaching the interior of exposed coral reefs would be a technical challenge. Box coring or direct sampling would result in resuspension of cuttings and an underestimation of concentrations. ‘Slurp gun’ sampling would result in either under or over estimations, depending on flow conditions within the reef at point of sampler application and the physical structure of the reef. Sediment trap sampling is likewise fraught with under and over estimation problems depending on trap design, and if employed as part of a monitoring strategy, trap designs would have to account for the tide and flow related turbidity loops which are often in evidence at reef sites [77].

#### Associate fauna

The macrofauna faunal communities observed at the various reefs were much as previously reported from coral reefs on the Norwegian margin [55,15]. Where sessile fauna commonly associated with CWC reefs were observed in images, their abundance did not change during the monitoring period, regardless of modelled particle exposure concentration. It has been argued that the commonly erect *Paragorgia arborea* coral prefers a more labile food supply than the less vertically extensive *Primnoa resedaeformis*, a growth mode more regularly exposed to refractory, resuspended material in high concentration [78]. In this study however both species seem unaffected by exposures to drill cutting plumes at the concentrations reaching the reefs, with no indications of colony mortality apparent in any images from any of the surveyed reefs.

The sessile filter feeding bivalve *Acesta excavata*, assumed to feed predominantly on very fine suspended material [79] did not appear to suffer from exposure to what was modeled to be environmentally high concentrations of fine drill cuttings either. Reef M17, where *Acesta excavata* were in high abundance before drilling, showed a comparable abundance >1 year after drilling. During drilling, during all ROV survey dives, the majority of *Acesta excavata* visible in video data (and extracted still images) were clearly open, regardless of whether particles were present in suspension or not (data not shown).

#### Increase in visible fragments on seafloor

Over time, the numbers of visible coral fragments surrounding some of the reefs did increase. The direct cause of this is not clear from this study. It seems unlikely that freshly observable fragments only a few weeks into drilling were the result of bioerosion of recently dead coral skeletons, given that such a process takes a minimum of several years [67] It is possible that plumes of resuspended material in the area resulting from the deployment of anchor blocks prior to drilling were transported to the monitored reefs in advance of monitoring. Possibly this settled material was then resuspended during periods of high benthic flow, (such as the high flow associated with the storms which interfered with drilling and damaged the data buoy initially used for real-time video monitoring of polyp activity) [30].

What is clear from comparison of the collected images is that where suitable images were taken, there is no increase in fragments on the seafloor in the >1 year images, at any of the reefs, as might be expected if polyps had been killed by drill cutting exposure and later bioeroded.

### 4.3 Visual validation of the modelling results

Though the visual assessment of particle concentrations in suspension during the ROV video surveys was categorical and open to observer bias, they did indicate that modeled particulate concentrations increased with increase in manual assessment of particle densities in the water column (Table 5). ROV dive plans made no attempt to film each reef for the full hour mirroring the output from the model runs. Much more commonly an ROV would briefly film a reef for a few minutes before landing and zooming the cameras in on a section of coral or climbing higher into the water column and proceeding to the next reef target. As Fig 8 shows, change in visual concentration of particles at a reef (and therefore actual ppm in suspension) can occur over a few seconds during drilling operations. The dive plans could therefore have missed high concentration plumes of particulates modeled to occur within a particular hour. The results presented here are promising, supporting the validity of the modeling approach in determining drill cutting dispersal in the reef environment, and this investigation should ideally be repeated with longer video monitoring or time lapse photography of reef locations during a modeled dispersal, to acquire a more consistent dataset from which to draw stronger conclusions on model validity. Investigating the validity of modelled dispersal predictions is becoming progressively more important to legislators [80, 81] and operators on the Norwegian margin, with work ongoing to assess the applicability of various modelling approaches [82].

### 4.4 Constraints of methodologies employed

#### Video data quality and consistency

Monitoring impacts of drill cuttings in the deep sea is challenging, even in regions with a homogeneous seafloor. In areas of high complexity, monitoring designs based on species abundance analysis from video transect data, such as recently completed for off-reef areas of the Morvin field [29] are not applicable. Direct sampling of the reefs by box core is too invasive and the technique would likely alter reef functioning to a greater extent than exposure to suspended materials could, with locations of physical damage opening the reef structure to fungal and sponge attack [67] as well as decreasing reef physical stability. All monitoring approaches must be predominantly photographic / video based in the future. A complementary method to such visual analysis, to determine exposure levels in-situ more accurately, would be the deployment of in situ pumps, turbidity meters or lazer particle sizers upstream and downstream of a reef of interest. Turbidity meters and particle sizers can be run in real time if connected by cable to a monitoring station but collected data would not record material composition, as is possible with pump systems. Without the composition data, extracting the drill cutting contribution of the total suspended particle load is not possible from particle sizer or turbidity meter data, although a long period of data recording prior to drilling (a control of sorts) would be beneficial in this. At present, there are the first attempts being made to provide real-time measurements of particle transport in the vicinity of drill cutting release and coral reefs [53]—a trend which will likely continue in the future and be of significant use to regulating bodies [22].

In this study the aim was to visually assess whether or not exposure to drill cuttings (at different concentrations and for different durations of time) would impact on *Lophelia pertusa* health. Although ROV video data was collected from a number of reefs exposed to different concentrations of material, comparisons in *Lophelia pertusa* behavior and changes in reef form over time was made highly problematic by the use of different ROVS, lighting rigs, cameras, zoom settings and piloting strategies on the various dives. The deployment of fixed reef markers allowed roughly comparable areas of reef to be filmed wide angle throughout the monitoring period, although viewing orientation differed between visits. These differences in orientation rendered observations of coral breakage or growth within reef structures very difficult. Suitable video data can be collected by ROV, if a rigorously repeated video collection regime is employed through successive visits [83]. The close up video data for comparison of polyp activity between reefs and over time was likewise variable in quality, with differences in illumination having perhaps the largest effect on clarity of collected data.

Fixed cameras have been mounted on lander systems for collection of suitable image data for time series analysis for decades. Whilst these Lander systems are useful in homogenous areas, they are less useful in areas such as coral reefs where the high spatial habitat variability requires collection of data from many points to adequately gauge change following environmental perturbation. In such environments, stable, mobile Crawler platforms may be applicable, with fixed illumination and camera mountings, coupled with the facility to move as required from monitoring location to monitoring location in rapid succession [84]. Re-deployable lander systems are a less attractive option as they require substantial cables from seafloor to surface to allow retrieval and repositioning of the Lander system, as well as a manned surface vessel, both of which may interfere with drilling operations.

#### Lack of background observation data

In this monitoring campaign reefs were visited by ROVs prior to the commencement of drilling operations, with video data collected from 9 individual reefs. This pre-drilling data however does not reflect the ‘pristine’ reef situation, as local perturbation and resuspension of the seafloor may have been high during rig deployment. It cannot be wholly discounted that the behavior patterns of coral polyps recorded in this study were not in some way different during the pre-drilling surveys than they would have been should the drill rig not have been deployed.

During the planning of the monitoring campaign, reef M17 was envisaged to be likely exposed to zero or negligible drill cutting concentrations. In the event, the benthic flow conditions experienced during the drilling campaign were not as predicted from advance survey, and reefs MA2 and MA3 were modeled to be those which likely experienced the least exposure. In any case, all the reefs, including MA2, MA3 and M17 were situated within the anchor corridors of the drill rig and were possibly subject to exposure from plumes of resuspended seafloor material prior to the commencement of the video monitoring survey. A more suitable reference reef would be one situated at similar depth several km from the end of the planned anchor corridors. Unfortunately, due to the availability of ROVs for the current study, the monitoring of such a distant location over time was not possible. Future studies should ideally monitor such control reefs, both in the short term during drilling and for later comparison with exposed reefs after several years.

### 4.5 Gaps in data

Whilst this study is the first to collect video data prior, during, immediately after and >1 year after drilling from reefs close to a drill site, the study is still of short duration when the life cycle of *Lophelia pertusa* is considered—an assumed life expectancy of an individual coral animal is ~30 years, with the fastest growth taking place during the first 5 years of a polyp’s life [41]. Even if higher quality, more comparable video data had been collected from each of the surveyed reefs during this monitoring period there would be little chance of observing significant differences in coral growth rates between reefs exposed to plumes of drill cuttings and those unexposed over this short duration monitoring period.

### 4.6 Further considerations

A visual inspection of an established reef prior, during and after an anthropogenic perturbation of the environment is critical when trying to establish the extent and / or severity of impact events. The negative impacts the *Deepwater Horizon* accident had on CWC communities in the Gulf of Mexico could be placed in context given previous investigations of reefs in the area [85]. Such studies however do not cover all possible impacts. Though in the current study exposure to drill cuttings, during and following the drilling period seems to have had minimal impact on adult *Lophelia pertusa* polyps and other sessile macrofauna—impacts other categories of reef fauna or *Lophelia pertusa* larvae, if present. For *Lophelia pertusa* (and many other CWC associate species) there are unanswered questions on the reproductive cycle and larvae transport. Though likely an annual broadcast spawning species [71, 86] there are many open questions on the stages of reproduction and larval dispersal. Though only a few larvae were available for experimental study, preliminary work indicates that *Lophelia pertusa* larvae may be highly sensitive to even environmentally low concentrations of suspended material, easily destroyed by contact [36]. It is possible that certain reefs in European seas are responsible for distributing larvae over considerable distances [87] and the release of drill cuttings into waters in the vicinity of reefs during spawning events may have consequences over wider areas than the few kilometers surrounding a drilling rig.

### 4.7 Overall conclusions

This study presents the observations made prior, during and after drill cutting release during a drilling campaign within the Morvin field, Norway. Opportunistic ROV video survey data was used to attempt the visual monitoring of reefs over time to assess any negative impacts of low particle concentration exposures and to validate modelled dispersal predictions. The video data were not collected in a standardized fashion, despite the deployment of reef markers to aid in this, and a range of dive protocols and cameras were used, making cross-comparison problematic. Video data was of sufficient quality to show that for the concentrations of drill cuttings to which the reefs were exposed, no visible impacts were apparent on surveyed megafauna and no sizable deposits of settled material were evident. This study did not take into account the possible negative effects exposure to drill cuttings may have had on larvae from corals or other reef species. There appeared to be a correlation between predicted peaks in drill cutting transport and visual observations of suspended material, though video data were only collected periodically from the reefs and therefore this observation should be treated with some caution. Further, this study investigated reefs less than two years after drilling, and any longer term impacts would therefore not be apparent. A further follow up study is recommended. Finally, the ROV video observations were only of sufficient quality for impacts on larger megafauna to be gauged. Any potential impacts on smaller fauna, such as small filter feeding sponges, hydroids etc. could not be determined. Impacts on mobile potentially commercial species of fish [88] were also not investigated here.

## Supporting Information

S1 File Sediment plume passing through reef ecosystem video. Video showing arrival of short duration turbidity pulses at reef following drill cutting release. Video collected by stationary ROV, with plumes not resuspended material resulting from ROV operations.
(WMV)
